# Discomfort and factual recollection in intensive care unit patients

**DOI:** 10.1186/cc2976

**Published:** 2004-10-28

**Authors:** Johannes P van de Leur, Cees P van der Schans, Bert G Loef, Betto G Deelman, Jan HB Geertzen, Jan H Zwaveling

**Affiliations:** 1 Physiotherapist, Center for Rehabilitation, University Hospital Groningen, Groningen, The Netherlands; 2 Professor of Nursing Science, University for Professional Education, Hanzehogeschool Groningen, and Department of Health Sciences, University of Groningen, Groningen, The Netherlands; 3 Supervisor, Department of Cardio-Thoracic Surgery, University Hospital Groningen, Groningen, The Netherlands; 4 Emeritus Professor of Neuropsychology, Department of Neuropsychology, University Hospital Groningen, Groningen, The Netherlands; 5 Professor of Rehabilitation, Center for Rehabilitation, University Hospital Groningen, and Northern Center for Health Care Research, Groningen, The Netherlands; 6 Professor of Surgical Intensive Care, Department General Surgery and Surgical Intensive Care Unit, University Hospital Groningen, Groningen, The Netherlands

**Keywords:** discomfort, hallucinations, intensive care unit, recollection

## Abstract

**Introduction:**

A stay in the intensive care unit (ICU), although potentially life-saving, may cause considerable discomfort to patients. However, retrospective assessment of discomfort is difficult because recollection of stressful events may be impaired by sedation and severe illness during the ICU stay. This study addresses the following questions. What is the incidence of discomfort reported by patients recently discharged from an ICU? What were the sources of discomfort reported? What was the degree of factual recollection during patients' stay in the ICU? Finally, was discomfort reported more often in patients with good factual recollection?

**Methods:**

All ICU patients older than 18 years who had needed prolonged (>24 hour) admission with tracheal intubation and mechanical ventilation were consecutively included. Within three days after discharge from the ICU, a structured, in-person interview was conducted with each individual patient. All patients were asked to complete a questionnaire consisting of 14 questions specifically concerning the environment of the ICU they had stayed in. Furthermore, they were asked whether they remembered any discomfort during their stay; if they did then they were asked to specify which sources of discomfort they could recall. A reference group of surgical ward patients, matched by sex and age to the ICU group, was studied to validate the questionnaire.

**Results:**

A total of 125 patients discharged from the ICU were included in this study. Data for 123 ICU patients and 48 surgical ward patients were analyzed. The prevalence of recollection of any type of discomfort in the ICU patients was 54% (*n *= 66). These 66 patients were asked to identify the sources of discomfort, and presence of an endotracheal tube, hallucinations and medical activities were identified as such sources. The median (min–max) score for factual recollection in the ICU patients was 15 (0–28). The median (min–max) score for factual recollection in the reference group was 25 (19–28). Analysis revealed that discomfort was positively related to factual recollection (odds ratio 1.1; *P *< 0.001), especially discomfort caused by the presence of an endotracheal tube, medical activities and noise. Hallucinations were reported more often with increasing age. Pain as a source of discomfort was predominantly reported by younger patients.

**Conclusion:**

Among postdischarge ICU patients, 54% recalled discomfort. However, memory was often impaired: the median factual recollection score of ICU patients was significantly lower than that of matched control patients. The presence of an endotracheal tube, hallucinations and medical activities were most frequently reported as sources of discomfort. Patients with a higher factual recollection score were at greater risk for remembering the stressful presence of an endotracheal tube, medical activities and noise. Younger patients were more likely to report pain as a source of discomfort.

## Introduction

Being admitted to an intensive care unit (ICU) can be considered a stressful life event, the reason for admission being a critical or even life-threatening condition. The ICU stay itself may also be stressful. Some patients report vivid recollections [1-3] whereas others have a poor or even no recollection at all of their stay on the ICU. No recollection at all of the ICU study ranges from 23% to 38% among postsurgical patients [4]. Various authors have reported that patients had unpleasant recollections after a stay on an ICU. Patients recalled discomfort arising from anxiety, pain, thirst, sleeplessness, disorientation, shortness of breath, inability to move, painful medical interventions and the presence of an endotracheal tube [5]. Turner and coworkers [6] specifically mentioned arterial blood gas sampling and endotracheal suctioning.

However, recollection of discomfort during the ICU stay is inseparably connected to the quality of recollection itself: events considered stressful at the time may not be remembered; conversely, recollections of stressful events may not be based on actual experiences. Jones and coworkers [7] investigated patients' estimation of the duration of their ICU stay in order to evaluate the accuracy of their memories. The patients' recall of events was generally poor, and 41% of them felt that they had been confused at some time during their stay in the ICU. To our knowledge, there is no literature investigating whether the recollection of discomfort is related to the accuracy of recollection of facts as such, and for what sources of discomfort this holds true. The purpose of this study was to describe the incidence of discomfort reported by ICU patients, the sources of their discomfort, the factual recollection of ICU patients and ward patients, and determinants of the recollection of discomfort in ICU patients.

## Methods

Consecutive ICU patients, who were older than 18 years and who had undergone intubation for longer than 24 hours, were included in the study. During mechanical ventilation patients received sedation by continuous infusion of midazolam (range 1–4 mg/hour) and fentanyl (range 50–150 µg/hour), with the degree of sedation given depending on their clinical requirements. The patients participated in a study comparing routine endotracheal suctioning with minimally invasive airway suctioning. The study was approved by the medical ethics committee of the University Hospital. The Acute Physiology Age and Chronic Health Evaluation (APACHE) II score was used to quantify the severity of illness [8] and was recorded on the day of admission to the ICU.

All ICU patients participated in a structured in-person interview, using a standardized questionnaire, within three days after discharge from the ICU to the ward. The reference group consisted of postsurgical ward patients, matched for age and sex. Data from the reference group were obtained in a structured telephone interview conducted within three days after discharge from hospital. In the questionnaire, all patients were asked to give answers to 14 questions concerning the ICU environment (lighting, timing of ward rounds, number of fellow ICU patients), the nursing staff (uniform, male/female) and personal care (clothing, position of intravenous drip, washing and toilet activities).

Patients from the ICU group were asked whether they remembered any discomfort during their stay on the ICU, and if they did then they were asked to specify the sources of discomfort that they remembered.

The questions regarding recollection of facts were first asked as open questions. Two points were given for each correct answer to these open questions. Patients who were unable to answer the open questions were presented with four multiple choice answers. One point was given for each correct answer to the multiple choice questions. Summation of the points resulted in a total score for factual recollection, providing an indication of the level of factual recollection. The range for the total score was 0–28 points.

### Statistical analysis

SPSS version 10 (SPSS Inc., Chicago, IL, USA) was used to perform all analyses. To assess the reliability of the questionnaire, a Cronbach's alpha was calculated. Differences between the ICU group and the reference group were analyzed using the ?^2 ^test for categorical variables and the t-test for normally distributed intervals or ratio scale variables. Differences between patients who recalled discomfort and those who recalled no discomfort were analyzed using the ?^2 ^test in case of categorical variables, the Mann–Whitney test for ordinal variables and the t-test for normally distributed intervals or ratio scale variables such as age. To analyze potential determinants of discomfort, logistic regression was performed. The presence or absence of discomfort was entered as the dependent variable, and independent variables were as follows: age, sex, APACHE II score (only in ICU patients), length of stay in the ICU or ward, factual recollection score and duration of tracheal intubation. Correlation coefficients between factual recollection score and age were calculated using a Spearman's test for categorical variables.

From the logistic regression analysis, odds ratios (ORs) were calculated for all independent variables in the equation. The OR expresses the odds in the group with the condition relative to the other group without the condition. To an extent, the OR can be considered a measure of relative risk. An OR greater than 1 indicates a higher risk and an OR below 1 indicates a lower risk in the group with the condition relative to the group without the condition.

## Results

A total of 125 patients discharged from the ICU were included in this study. Two patients were unable to respond to the questions. Patient characteristics are summarized in Table 1. In the population studied the prevalence of any discomfort recalled after discharge from the ICU was 54% (*n *= 66). The sources of discomfort identified by these 66 patients are summarized in Table 2. Six patients were disorientated at the time of the interview, but were able to recall discomfort.

The median (min–max) factual recollection score was 15 (0–28) in the ICU patients and 25 (19–28) in the reference group; the difference between the groups was highly significant (*P *< 0.001). Analyses of reliability of the questionnaire for the ICU patients revealed a Cronbach's alpha of 0.86, indicating high reliability. Items of factual recollection by ICU patients and the reference group, in descending order of being identified correctly, are listed in Table 3.

ICU patient characteristics are summarized in Table 4 separately for the group that recalled any discomfort and the group that did not recall any discomfort. Significant differences were found between the two groups in factual recollection, age and duration of intubation.

Logistic regression analysis of determinants of recollection of discomfort confirmed that factual recollection was indeed an independent factor in predicting recollection of discomfort. The calculated OR was 1.1 (*P *< 0.001), with a correct percentage in regression analysis of 68%.

This implies that the risk for recalling discomfort was 1.1 times higher for each factual recollection point. Age also was a determinant of recollection of discomfort. The calculated OR was 0.97 (*P *= 0.006; correct percentage in regression analysis 66%). This implies that the risk for recalling discomfort was lower by a factor of 0.97 for each year of advancing age. The duration of intubation appeared not to be independently related to recollection of discomfort.

**Figure 1:**
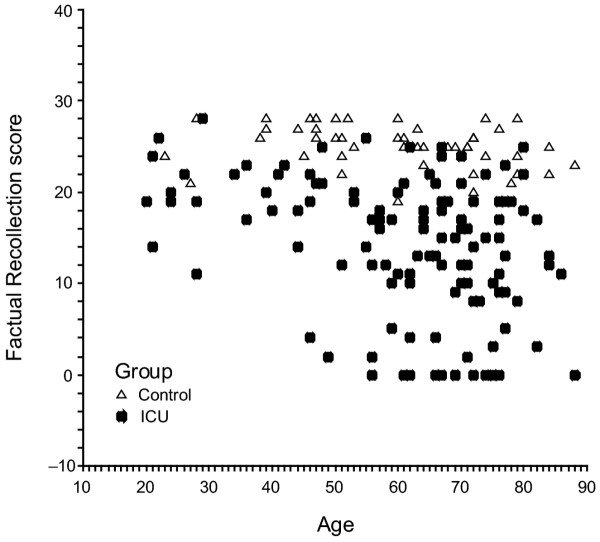
Scatterplot of factual recollection by age in intensive care unit (ICU) patients and the reference group (Control).

Factual recollection appears to be inversely related to age. Analysis of the relationship between factual recollection score and age in the ICU group revealed that the correlation coefficient was -0.352 (*P *< 0.001); in the reference group it was -0.327 (*P *= 0.023; Fig. 1).

Finally, recollection of pain appeared to be related to age (OR 0.936, *P *= 0.002; correct percentage in regression analysis 94%). This implies that younger patients reported more recollection of discomfort in the form of pain.

## Discussion

The results of the present study show that a considerable proportion (54%) of patients discharged from the ICU had a recollection of discomfort during their stay in the ICU. The presence of an endotracheal tube, medical interventions, noise and experiences of hallucination were among the sources of discomfort most frequently reported. To our knowledge, this study is the first to evaluate the association between recollection of discomfort and intact factual recollection. In a study conducted by Rose and coworkers [9] in 50 patients, 60% remembered endotracheal suctioning and 52% remembered extubation as unpleasant experiences. In a study by Turner and coworkers [6], arterial blood gas sampling and tracheal suctioning were recalled by 48% and 44% of the patients. Although those two studies did not investigate the prevalence of discomfort *per se*, we conclude that their findings are similar to ours, in that discomfort was recalled by 54% of ICU patients.

Within the context of ICU patients' recollections, a memory of an (stressful) event raises the question of whether this recollection is based on reality or fantasy/imagination. In the present study we found the degree of factual recollection to be an important determinant of discomfort, in the sense that more discomfort was reported by those with better factual recollection. Each item of factual recollection that was scored correctly increased slightly the risk for recollection of discomfort. Factual recollection and recollection of discomfort therefore appeared to be related.

In an ICU many factors contribute to impairment in memory: critical illness itself, the use of benzodiazepines and opioids, and the common occurrence of delirious states. When a patient's health is improving or when sedative agents are reduced below effective levels, patients tend to remember more regarding factors, mostly unpleasant, in the ICU. Jones and coworkers [10] described many causes of amnesia during severe illness, including large dosages of sedative medication and withdrawal syndromes. Because levels of sedation strongly influence the function of memory, a weak point in our study is that no sedation score was recorded to enable us to evaluate the effects of sedatives on patient recollection. It should also be noted that we did not look for objective signs of postdischarge psychological distress or examine their relationship to memories of stressful events, either real or perceived. We merely wished to improve our understanding of discomfort by taking into account the confounding role of memory.

The presence of an endotracheal tube, medical activities, and noise and bustle were the sources of discomfort remembered most frequently (Table 2). This finding is comparable with those of other studies. In a group of 68 ventilated medical patients, Turner and coworkers [6] found a prevalence of recollection of endotracheal suctioning of 44%, and in 26 mainly surgical patients those investigators found a prevalence of recollection of endotracheal suctioning of 47% [11]. In a mixed surgical/medical group of cardiac patients (*n *= 50), Rose and colleagues [9] found a 60% prevalence of recollection of endotracheal suctioning during the ICU stay.

The reason for discomfort relating to the endotracheal tube may be endotracheal suctioning. While intubated, patients are regularly suctioned via the endotracheal tube in order to maintain airway patency. The strong mechanical stimuli resulting from endotracheal suctioning may explain why the endotracheal tube is remembered as a prominent source of discomfort. In a previous study [12], we investigated recollection of endotracheal suctioning with two methods of suctioning: routine endotracheal suctioning and minimally invasive airway suctioning. In the case of routine endotracheal suctioning, a 49 cm suction catheter was passed into the lower airways. With minimally invasive airway suctioning the suction catheter did not enter the lower airways and suctioning was limited to the endotracheal tube. A significantly lower prevalence of recollection of airway suctioning was found in the minimally invasive airway suctioning group (20%) than in the routine endotracheal suctioning group (41%; *P *< 0.001). Our findings show that discomfort resulting from the endotracheal tube and its handling can be reduced by changing the procedure.

Hallucinations were another source of discomfort. In the total ICU patient group (*n *= 123), 24 (20%; 95% confidence interval 13–23%) of patients experienced hallucinations. This finding is comparable with that of an earlier and smaller study conducted by Holland and coworkers [2], who found that 10% of patients reported hallucinations. In a more recent study, Ely and colleagues [13] found that 81.7% of ICU patients developed delirium at some stage in their ICU stay. Delirium was an important variable, contributing as an independent predictor to higher 6-month mortality and longer hospital stay. Delirium was defined as 'a disturbance in consciousness characterized by an acute onset and fluctuating course of impaired cognitive functioning so that a patient's ability to receive, process, store and recall information is strikingly impaired'. Clearly, the presence of delirium by this definition does not imply the presence of hallucinations. The exact percentage of patients who recalled hallucinations was not stated in the report by Ely and coworkers.

In studies conducted by Puntillo [14] and Holland and coworkers [2], pain was reported as a source of discomfort as well. In a post-cardiac surgery population (*n *= 24), Puntillo [14] described awareness of pain during the ICU period as a significant problem. Holland and coworkers [2] reported that, in a group of postsurgery patients (*n *= 21), 71% had a recollection of pain. In our study of mainly surgical ICU patients, only 12% indicated that pain was a source of discomfort. Differences in type of sedation and pain medication, number of patients, inclusion criteria and type of questionnaire used are possible explanations for the low recollection of pain in the present study as compared with previous ones.

A standardized score to assess recollection in this type of patient was lacking at the time our study was performed. We developed a factual recollection questionnaire that may represent a reliable new tool for acquiring information regarding recollection of facts in post-ICU patients. Analysis of reliability revealed a high Cronbach's alpha, and the descriptive data of our score showed a significant difference between ICU patients and the reference group. These findings are hardly surprising in view of the considerable differences between groups in severity of illness and consumption of hypnotics and sedatives. Further studies are needed to determine the validity and reliability of this instrument. Jones and coworkers [15] have since proposed a similar tool (Intensive Care Unit Memory tool), which has been validated in a number of settings [4,16].

Both good factual recollection and younger age increased the risk for discomfort. Factual recollection and age were inversely associated with each other, but this association was weak. The association of increasing age with reduction in memory function is widely recognized [17,18].

Although factual recollection and recollection of discomfort appear to be related, increasing the level of sedation is not necessarily the best way to prevent discomfort. Not only will deep sedation lead to increased length of stay in the ICU and prolonged ventilator dependency [19] but it may also have an adverse effect on the rate of post-traumatic stress disorder experienced by patients after their discharge from the ICU [10]. It has been proposed by various authors that factual recollection helps to offset the emotional impact of delusional memories [10,19] and may actually help to avoid adverse psychological outcomes in this type of patient. The development of drugs that can eliminate the emotional impact of stressful events in the ICU, while preserving mental clarity and memory, might offer the best way to avoid long-term psychological distress. Meticulous treatment of delusional states will also contribute to this end.

## Conclusion

In a series of patients discharged from the ICU, 54% recalled discomfort. The most frequent sources of discomfort cited were presence of an endotracheal tube, hallucinations and medical interventions. The median factual recollection score for ICU patients was significantly lower than the median factual recollection score for ward patients who had not been in an ICU environment. Younger patients were at greater risk for remembering pain as source of discomfort. Patients with better factual recollection had greater recollection of discomfort. Factual recollection and age were inversely related, but this relationship was weak.

Discomfort thus appears to be a serious problem for patients in an ICU environment. Its prevalence is probably underestimated because retrospective assessment of the degree of discomfort when the patient has been discharged from the ICU is seriously handicapped by global or partial amnesia, caused by critical illness, delusional states and the use of drugs. However, the fact that discomfort is not always remembered does not imply that the patient has not suffered during his or her stay in the ICU. Reduction in discomfort should remain a focus of attention for both researchers and clinicians caring for critically ill patients.

## Key messages

• 	Discomfort is a serious problem; 54% of ICU patients experienced discomfort.

• 	Endotracheal tube, hallucinations and medical interventions were cited as sources of discomfort.

• 	Patients with a higher factual recollection have greater recollection of discomfort.

## Abbreviations

APACHE = Acute Physiology and Chronic Health Evaluation; ICU = intensive care unit; OR = odds ratio.

## Competing interests

The author(s) declare that they have no competing interests.

## Author's contributions

JvdL designed the study, performed data collection, data entry, statistical analysis and wrote the manuscript. CvdS, BL, BD and JZ participated in the design of the study. CvdS, BL, JG and JZ participated in the statistical analysis and writing the manuscript.

## Figures and Tables

**Table 1:** Patient characteristics

Characteristic	ICU group (*n *= 123)	Reference group (*n *= 48)	*P*
Age (mean ± standard deviation)	61.5 ± 16	60.2 ± 16	0.617
Male sex (%)	71	65	0.435
APACHE II score (median [min–max])	11 (2–26)	NA	-
Type of patient: trauma/medical/surgical (%)	8/7/85	13/4/83	0.537
ICU stay in days (median [min–max])	6.5 (2–133)	NA	-
Ward stay in days (median [min–max])	NA	10 (3–53)	-

APACHE, Acute Physiology and Chronic Health Evaluation; ICU, intensive care unit; NA, not applicable.

**Table 2:** Sources of discomfort in intensive care unit patients (*n *= 66)

Source of discomfort	%^a^
Endotracheal tube	42
Hallucinations	32
Medical activities	29
Noise and bustle	14
Pain	12
Thirst	9
Inability to talk	9
Shortness of breath	6
Being afraid	6

^a^Because patients could list more than one source of discomfort, the summation of percentages exceeds 100%.

**Table 3:** Items of factual recollection by intensive care unit patients and the reference group

	Correct (%)		Incorrect (%)		Don't know (%)	
	
Factual recollection	ICU group	Reference group	ICU group	Reference group	ICU group	Reference group
Type of patients' clothing^†^	68	100	12	0	20	0
Gender of nursing staff^†^	66	98	7	2	27	0
Place of intravenous access^†^	65	98	11	0	24	2
Color of staff uniform^†^	62	98	14	2	24	0
Number of fellow patients*	62	71	8	17	30	12
Type of personal hygiene^†^	62	98	7	2	31	0
Logo on staff uniform^†^	55	88	5	0	40	12
Type of lighting†	54	96	12	4	34	0
Reason inability to talk^†^	50	94	24	6	26	0
Time of personal hygiene^†^	48	100	3	0	49	0
Toilet visits†	42	100	32	0	26	0
Alternative headstand positions of bed^†^	42	92	18	6	40	2
Type of food received^†^	23	100	54	0	23	0
Time of ward round^†^	11	98	34	0	55	2

*P < 0.05 and ^†^*P *< 0.005, intensive care unit patients versus reference group, by ?^2 ^test.

**Table 4:** Characteristics of ICU patients with and those without recollection of discomfort

Characteristic	Discomfort (*n *= 66)	No discomfort (*n *= 57)	*P*
Age (years; mean ± standard deviation)	59 ± 17	65 ± 14	0.004
Male sex (%)	65	77	0.143
APACHE II score (median [min–max])	12 (2–26)	11 (5–24)	0.171
Duration of intubation (days; median [min–max])	5 (2–35)	3 (1–57)	0.001
Factual recollection score (median [min–max])	18 (0–28)	11 (0–24)	<0.001

APACHE, Acute Physiology and Chronic Health Evaluation.

## References

[B1] Rundshagen I, Schnabel K, Wegner, Schulte am Esch J (2002). Incidence of recall, nightmare, and hallucination during analgosedation in intensive care. Intensive Care Medicine.

[B2] Holland C, Cason CL, Prater LR (1997). Patients' recollection in critical care. Dimens Crit Care Nurs.

[B3] Rotondi A, Chelluri L, Sirio C, Mendelsohn A, Schulz R, Belle S, Im K, Donahue M, Pinsky Mr (2002). Patients' recollection of stressful experiences while receiving prolonged mechanical ventilation in an intensive care unit. Crit Care Med.

[B4] Capuzzo M, Pinamonti A, Cingolani E, Grassi L, Bianconi M, Contu P, Gritti G, Alvisi R (2001). Analgesia, sedation and memory of intensive care. J Crit Care.

[B5] Pennock BE, Crawshaw L, Maher T, Price T, Kaplan PD (1994). Distressful events in the ICU as perceived by patients recovering from coronary artery bypass surgery. Heart Lung.

[B6] Turner JS, Briggs SJ, Springhorn HE, Potgieter PD (1990). Patients' recollection of intensive care unit experience. Crit Care Med.

[B7] Jones J, Hoggart B, Withey J, Donaghue K, Ellis BW (1979). What the patients say: a study of reaction to an intensive care unit. Intensive Care Med.

[B8] Knaus WA, Draper EA, Wagner DP, Zimmerman JE (1985). APACHE II: a severity of disease classification system. Crit Care Med.

[B9] Rose D, Roggla M, Behringer W, Roggla G, Frass M (1999). Recollections of ventilated patients after a stay in the intensive care unit [in German]. Wien Klin Wochenschr.

[B10] Jones C, Griffiths RD, Humphris G (2000). Disturbed memory and amnesia related to intensive care. Memory.

[B11] Turner JS, Messervy SJ, Davies LA (1992). Recollection of intensive care unit admission in the United Kingdom [letter]. Crit Care Med.

[B12] Van de Leur JP, Zwaveling JH, Loef BG, Van der Schans CP (2003). Patient recollection of airway suctioning in the ICU: routine versus a minimally invasive procedure. Intensive Care Med.

[B13] Ely EW, Shintani A, Truman B, Speroff T, Gordon SM, Harrell FE, Inouye SK, Bernard GR, Dittus RS (2004). Delirium as a predictor of mortality in mechanically ventilated patients in the intensive care unit. JAMA.

[B14] Puntillo KA (1994). Dimensions of procedural pain and its analgesic management in critically ill surgical patients. Am J Crit Care.

[B15] Jones C, Griffiths RD, Humphris G, Skirrow PM (2001). Memory, delusions, and the development of acute posttraumatic stress disorder-related symptoms after intensive care. Crit Care Med.

[B16] Capuzzo M, Valpondi V, Cingolani E, De Luca S, Gianstefani G, Grassi L, Alvisi R (2004). Application of the Italian version of the Intensive Care Unit Memory tool in the clinical setting. Crit Care.

[B17] Wegesin D, Jacobs DM, Zubin NR, Ventura PR, Stern Y (2000). Source memory and encoding strategy in normal aging. J Clin Exp Neuropsychol.

[B18] Yokota M, Miyanaga G, Yonemura K, Watanabe H, Nagashima K, Naito K, Yamada S, Arai S, Neufeld RW (2000). Declining of memory functions of normal elderly persons. Psychiatry Clin Neurosci.

[B19] Kress JP, Gehlbach B, Lacy M, Pliskin N, Pohlman AS, Hall JB (2003). The long-term psychological effects of daily sedative interruption on critically ill patients. Am J Respir Crit Care Med.

